# Simultaneous Acquisition of Ultrasound and Gamma Signals with a Single-Channel Readout

**DOI:** 10.3390/s21041048

**Published:** 2021-02-04

**Authors:** Muhammad Nasir Ullah, Yuseung Park, Gyeong Beom Kim, Chanho Kim, Chansun Park, Hojong Choi, Jung-Yeol Yeom

**Affiliations:** 1 Global Health-Tech Research Center, Korea University, 145 Anam-ro, Seoul 02841, Korea; nasir@korea.ac.kr (M.N.U.); parkcs@korea.ac.kr (C.P.); 2 Department of Bioengineering, Korea University, 145 Anam-ro, Seoul 02841, Korea; ii1238@korea.ac.kr (Y.P.); kgb95c@korea.ac.kr (G.B.K.); manngo@korea.ac.kr (C.K.); 3 Department of Medical IT Convergence Engineering, Kumoh National Institute of Technology, 350-27 Gumi-daero, Gumi 39253, Korea; 4 Interdisciplinary Program in Precision Public Health, Korea University, 145 Anam-ro, Seoul 02841, Korea; 5 School of Biomedical Engineering, Korea University, 145 Anam-ro, Seoul 02841, Korea

**Keywords:** gamma probe, ultrasound probe, hybrid systems, silicon photomultiplier (SiPM), nuclear medicine (NM)

## Abstract

We propose an integrated front-end data acquisition circuit for a hybrid ultrasound (US)-gamma probe. The proposed circuit consists of three main parts: (1) a preamplifier for the gamma probe, (2) a preprocessing analog circuit for the US, and (3) a digitally controlled analog switch. By exploiting the long idle time of the US system, an analog switch can be used to acquire data of both systems using a single output channel simultaneously. On the nuclear medicine (NM) gamma probe side, energy resolutions of 18.4% and 17.5% were acquired with the standalone system and with the proposed switching circuit, respectively, when irradiated with a Co-57 radiation source. Similarly, signal-to-noise ratios of 14.89 and 13.12 dB were achieved when US echo signals were acquired with the standalone system and with the proposed switching circuit, respectively. Lastly, a combined US-gamma probe was used to scan a glass target and a sealed radiation source placed in a water tank. The results confirmed that, by using a hybrid US-gamma probe system, it is possible to distinguish between the two objects and acquire structural information (ultrasound) alongside molecular information (gamma radiation source).

## 1. Introduction

Recent technical advancements and innovations in the field of surgical technologies have vastly improved the resection precision of diseased tissues such as tumors. The utilization of various types of intraoperative probe during surgical procedures has not only reduced the probability of a false positive but, also, the occurrence of postoperative complications [1,2]. Various well-established types of surgical probe are used for the intraoperative procedures. Among them, the nuclear medicine (NM) probes, which include the gamma probe and the positron probe, are widely used for sentinel lymph node and tumorous tissue localization via detection of the gamma radiation emitted by radiopharmaceuticals [3,4,5,6,7,8]. NM probes provide a high level of molecular information that is beneficial to diagnosis abnormalities and metabolic activity levels around the areas of interest [9]. Nevertheless, while NM probes can provide molecular-level information, it is often difficult to infer an intuitive link between the NM gamma radiation signals and the anatomical structure using this type of probe [10,11].

Therefore, the recent research trend revolves around developing a dual-modality detector and system to acquire both molecular and anatomical information simultaneously [9,10,12,13,14,15,16,17]. The incorporation of complementary information, such as anatomical and molecular information, has substantially improved the accuracy of the diagnosis and treatment procedures. Meanwhile, the exploration of the field of multi-model techniques has increased exponentially over the last decade. Examples of multi-model systems include the dual gamma-fluorescence probe, gamma-optical imager, and US-gamma probe [13,17,18,19]. Recently, a dual US-gamma integrated system was proposed [15,17], with the absence of additional radiation dose exposure and noninvasiveness making the US system an attractive choice for dual-modal systems.

Currently, information and images from the US and NM systems are acquired using separate standalone systems [20]. The total integration of the US and NM systems is challenging from the hardware point of view due to the differences in the sensor type, the readout electronics, and the signal-processing requirements [17]. While an integrated US-NM imaging probe was proposed [9], US and NM signals were acquired independently using separate front-end circuits for each system.

In this paper, we propose an integrated circuit for US-gamma probe readouts and data acquisition. Typical US and NM detectors are comprised of tens to hundreds of readout channels, meaning the proposed scheme could greatly simplify the data acquisition of combined US-NM systems. The proposed front-end circuit consists of preamplifiers for the gamma and US signals, and an analog filtering circuit. Meanwhile, an analog switch that can be digitally controlled is utilized to switch between the US and gamma signals, which means only a single readout channel is required for data acquisition and post processing. A single-channel US and customized gamma probe was used for the proof of the concept, which could have applications in non-imaging compact intraoperative probes or nondestructive tests (NDT), such as simultaneous thickness measurement using US and density measurement using a gamma detector [21,22,23]. The results demonstrate that an excellent performance is achieved for both gamma and the US probes while ensuring that the circuitry is integrated and compact.

This paper is organized as follows: In Section 2, the materials and method used for the assessment and validation of the proposed method are explained in detail. Section 2 is subdivided into three subsections. Section 2.1 discusses the gamma probe design and also includes brief discussion about the commercial Ultrasound probe used for this study. Section 2.2 consists of the proposed front-end circuit design for simultaneous read out of the dual US-gamma probe. The experimental setup and data acquisition system is covered in Section 2.3. The acquired experimental results for performance evaluation of both US and NM gamma probe are summarized in Section 3. Section 3 is also divided into three subsections, where the performances of the gamma probe, US probe, and the combined US-gamma probe are presented, respectively. Following the results, a discussion about the proposed circuit for dual US-gamma probe is presented in Section 4, and the paper ends with the conclusion in Section 5.

## 2. Materials and Methods

In this proof-of-concept study, a front-end electronic circuit was designed and tested to acquire both US and gamma signals simultaneously using a single readout channel. The basic principle behind the US system is to transmit a high-power US signal (transmit signals, TX) via a piezoelectric transducer and then to remain idle for a specific time period to allow for the detection of the reflected signals (receive signals, RX). This cycle is repeated at a predetermined frequency known as the pulse repetition frequency (PRF) [24,25,26]. However, the US system could remain in idle mode for a large portion of the time, depending on the PRF of the system and the imaging mode. Meanwhile, NM gamma radiations are received randomly. The proposed circuit thus utilizes the idle state of the US system to acquire the gamma signals. A digitally controlled analog switch was used for the readout of each sensor (US-gamma) based on the US transmit signal and on waiting for a specific time period for the receive signal.

**Figure 1:**
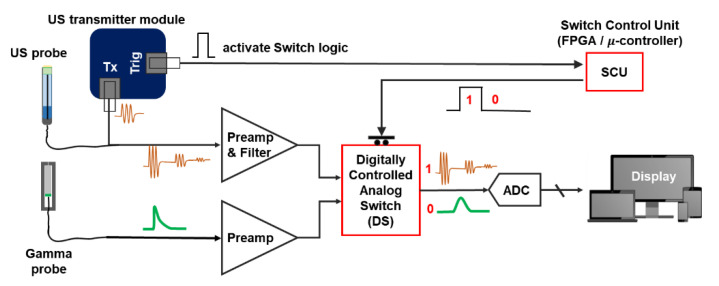
Schematic of proposed integrated US-gamma probe circuit for data acquisition using a single readout channel. See Section 2.2 for the detailed preamplifier design. A commercial US transmitter was used for this study. The transmitter generates the trigger signal at the time of transmit signal and that trigger signal is fed to the SCU (FPGA) to allocate the output channel to either US or gamma system for specific period of time.

The basic design of the method is illustrated in Figure 1. The basic components of the proposed data acquisition circuit include preamplifiers for both the US and gamma probes (see Section 2.2 for detail), an analog bandpass filtering circuit for US signal preprocessing, a switch control unit to generate the digital signal that is used to switch between US and gamma probe readout, and a digitally controlled analog switch (DS) to assign the respective probe to the analog-digital converter (ADC, high-speed oscilloscope).

Specifically, as the US system operates according to predetermined patterns (PRFs): the switch control unit (SCU) generates a “1” logic only for the duration required for US operation, i.e., a trigger signal (Trig) from the transmitter (pulser) module is used to activate the SCU and generate “1” logic for a specific time and “0” logic otherwise. Within the DS, an analog switch is used to connect the subsequent stage (ADC) to the US probe during “1” and gamma probe during “0”. Thus, in so doing, a single ADC is utilized to acquire data from both the US and the NM gamma probes. In this study, the field-programmable gate array (FPGA) was used as the SCU. However, a simple one-shot timer circuit or microcontroller can also be utilized as a SCU.

### 2.1. Gamma and Ultrasound Probe

In this study, two separate US and NM gamma probes were used. The two probes were then fastened to each other in the form of a hybrid US-NM probe for experimental purposes.

**Figure 2:**
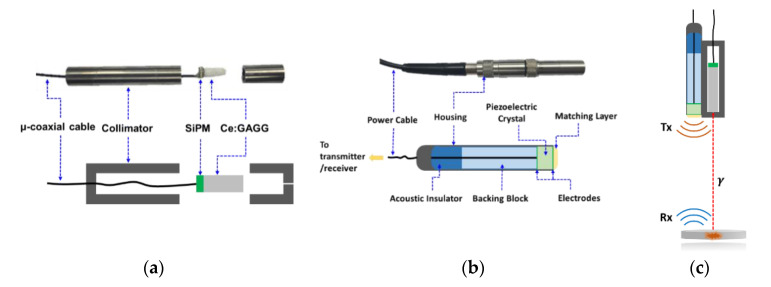
(**a**) Photograph and schematic of the nuclear medicine (NM) gamma probe and its components. (**b**) Commercial V311-SU 10 MHz transducer from Olympus Corp. used for US signal transmission and reception. (**c**) Illustration of the hybrid US-gamma probe.

The gamma probe was designed and constructed using a Ce:GAGG scintillation crystal with dimensions of 2 × 2 × 10 mm^3^. The scintillator was wrapped with Teflon tape, coupled to a silicon photomultiplier (SiPM) photodetector (Hamamatsu S13360-2050VE MPPC) with optical grease, and then inserted into a custom-designed tungsten collimator. A thin -coaxial-long cable was used to provide bias voltage to the SiPM and to transmit radiation signals to the electronics (Figure 2a). The diameter of the assembled NM gamma probe was 10 mm.

In terms of the US probe, a commercial V311-SU 10 MHz transducer from Olympus Corp. was used (Figure 2b). The diameter of the US probe was 13 mm.

### 2.2. Ultrasound and Gamma Analog Front-End Circuits

A single-channel front-end circuit was designed to acquire both US and gamma signals, as illustrated in Figure 1. The front-end circuit can be divided into three main parts: (1) a preamplifier circuit for the gamma signal, (2) a preamplifier and filtering circuit for US signal preprocessing, and (3) a switching circuitry to allocate the readout channel to each modality (US-gamma), along with a digital control for the switch.

**Figure 3:**
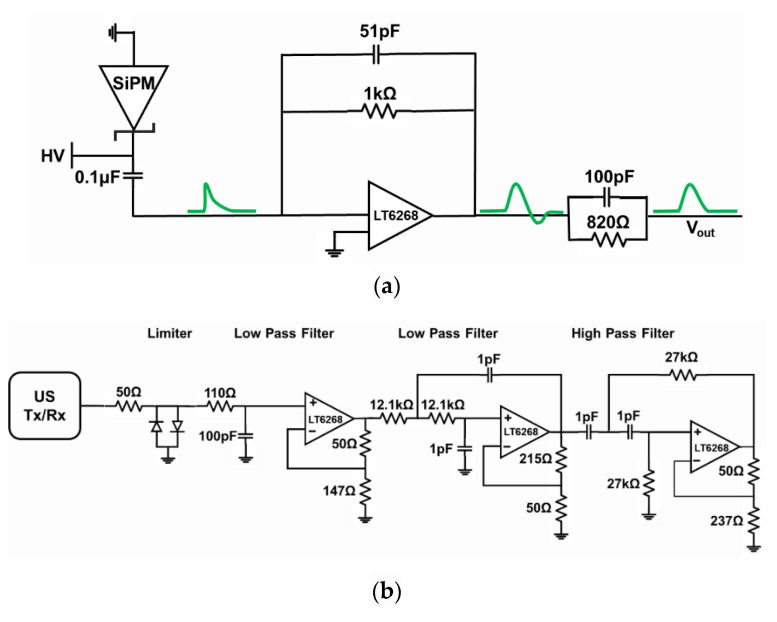
Schematic of an analog front end. (**a**) Charge-sensitive amplifier (CSA) front-end circuit for a NM gamma probe with pole-zero cancellation (100 pF and 820 Ω) to reduce the undershoot and (**b**) signal-processing circuit of the US probe.

In the case of a NM gamma probe, it is important to measure the energy of incoming radiation events (energy resolution). The shape and size of gamma signal vary depending on the type of scintillator and incident radiation energy. As the Ce:GAGG scintillator used in this study has a fast rise and decay time (~200 ps and 140 ns, respectively) [27], a charge-sensitive preamplifier (CSA) was implemented to integrate, shape (filter), and amplify the signal. By implementing a CSA, as opposed to a voltage amplifier, the peak value of the output signal (V_out_) represents the energy of the gamma signal. This allows data acquisition with a slow ADC. The schematic of the circuit is shown in Figure 3a. The gamma detector and CSA circuit gave rise to gamma radiation signals having a maximum value of about 0.47 V (with a Co-57 radiation source) and a center frequency of lower than 3 MHz. The noise level is lower than 0.01 V.

On the US side, a commercial system (Panametrics 9100) was used to create ultrasonic transmit pulses (TX) with the following settings: 120 V, 10 MHz 5-cycle sinusoidal waveform. In our setup, raw RX signals were ~0.08 V peak-to-peak and had a noise floor of around 0.004 V. These signals were fed to an active bandpass filter and amplification circuitry, as shown in Figure 3b. The values were selected such that signal components outside the 6.5~13.5 MHz frequency range were filtered away. Consequently, the 10-MHz TX signal is conserved while the noise is suppressed, and the echo signal is amplified ~13 times by the custom front-end circuit.

The heart of the proposed integrated front-end analog circuit is the switching circuit design. The speed of the US signal is around 1,450 m/s in human body fat. The human body’s cross-section length is, on average, 15 cm. Therefore, a US signal returning from the deepest point from inside the body can be reflected back in 200 μs after the transmit signal. As described above and illustrated in Figure 1, the DS (TS5A623157DGSR, Texas Instruments, Inc., Dallas, TX, USA) was controlled via the FPGA. The switch control unit was programmed such that, with every transmit signal (TX) from the US probe, a “1” is generated for 200 μs, and the switch allocates the output port to the US system for that duration. Otherwise, the switch connects the output to the gamma probe when “0” is generated.

### 2.3. Experimental Setup and Data Acquisition

**Figure 4:**
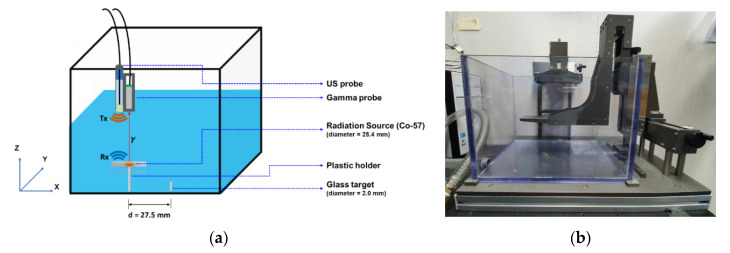
(**a**) Illustration of the experimental setup of the water tank in which a disk-type radiation source (Co-57) and a glass target were placed for experimental data acquisition. The targets were then scanned using the hybrid US-gamma probe. (**b**) Custom 3-axis moving stage for manipulating the hybrid probe across the water tank.

Several experiments were performed to assess and validate the performance of the proposed method. A small glass slab and a radiation source were placed in the water tank and then filled with double-distilled water. The radiation source was a disk-type sealed Co-57 radiation source, which was placed 27.5 mm away from the glass target (center to center), as illustrated in Figure 4a.

First, the performance (energy resolution) of the gamma probe was assessed separately without connecting the US probe and switching circuitry. Then, the energy resolution and the count rate (counts per second, cps) performance of the NM gamma probe were acquired using the proposed switching method, with different PRFs of the US transmitter. The data required to compute the energy resolution and count rate were obtained using a high-speed oscilloscope (Teledyne LeCroy, Chestnut Ridge, NY, USA, WaveRunner 604Zi) that can operate at a maximum sampling rate of 20 GS/s (400 MHz bandwidth). In our experimental setting, data were acquired at a 1-GS/s sampling rate. Nonetheless, a 250-MHz sampling rate ADC or less should suffice in our proposed system, considering the bandwidth requirements described above. Acquired data were then processed using a PC.

In the case of the US readout, although the commercial US system (Panametrics 9100) is able to provide transmit signals, as well as process echo signals, in this study, however, this commercial system was used to generate TX and Trig signals only. First, raw echo signals (RX) were acquired with an oscilloscope and processed in a PC. Next, signals were fed to our custom-designed readout front-end circuit and acquired. Lastly, both probes (US and gamma) were attached together in the form of a hybrid US-gamma probe. The results of these three tests are presented in the following subsection.

The water tank was then scanned along the *x*-axis across the center of the radiation source and glass target with a 1-mm step size. This was carried out using a manual 3-axis moving stage, shown in Figure 4b. The two probes (US (Olympus Inc., Shinjuku, Tokyo, Japan), NM gamma) were attached to the stage side-by-side (see Figure 2c), and the distance between the two probes was accounted for (to correct for the offset between the two probes).

## 3. Results

### 3.1. Gamma Probe Performance

Three different experiments were used to assess and evaluate the performance of the NM gamma probe using the proposed method. In the first setup (S1), data were acquired directly from the output pin of the gamma probe preamplifier while leaving the switching circuitry off to completely disconnect the US probe. This was conducted to assess the gamma probe as a standalone system and to check for any degradation in performance when the gamma probe was used in conjunction with the US probe and switching circuitry.

**Figure 5:**
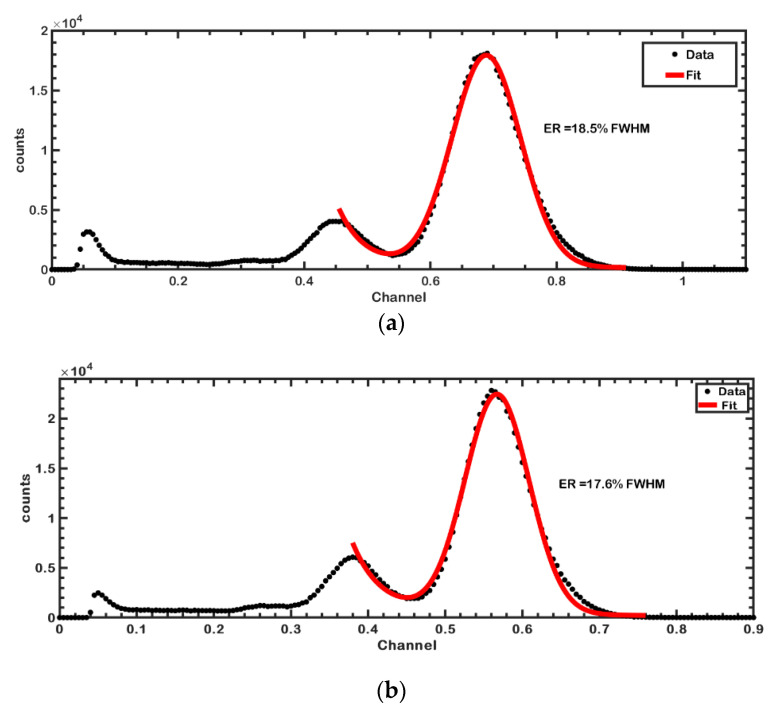
Energy spectra acquired using the Co-57 radiation source with the proposed gamma probe. (**a**) Energy spectrum and resolution (18.5%) acquired from the standalone gamma probe without using the integrated US-gamma probe front-end circuit (setup S1). (**b**) Energy spectrum and resolution (17.6%) when the proposed front-end integrated US-gamma probe circuit was used (setup S2).

Next, data were acquired from the output of the switching circuitry. In this setup (S2), the switching circuitry and US system were turned on. The energy spectra of the NM gamma probe were acquired, and the energy resolutions were computed. Energy resolutions of 18.5% and 17.6% full-width-half-maximums (FWHM) were acquired at the Co-57 photoelectric peak for the S1 and S2 setups, respectively (Figure 5a,b). It was clear that the switch and the US system had no effect on the energy performance of the gamma probe.

In the last experiment, the count rate performance (cps) was calculated while varying the PRF of the US system from 0–4 kHz. For each US repetition rate, gamma probe count rate data were acquired from the switch output for a one-minute time period. An energy window from 110–132 KeV was set to selectively obtain events that fall within the peak position, and the count rate (cps) was calculated, as summarized in Table 1. The count rate was found to decrease with an increase in US PRF (or duty factor) as more readout time was allocated to the US system.

### 3.2. Ultrasound Probe

The commercial US system (Panametrics 9100) was used only as a US pulser. The generated pulses were delivered to the commercial US probe (V311-SU), and the echo data was acquired using an oscilloscope. Three sets of US echo receive signals (RX) were acquired as follows: (1) raw RX signal from the US probe without any front-end circuit (setup S3), (2) custom-designed analog front-end readout circuit only (setup S4), and (3) custom-designed front-end readout circuit while turning on the switch and gamma probe (setup S5).

As Table 2 shows, our custom-designed US readout circuit provided a significant filtering and amplification performance. However, a slight degradation of the US probe signal was observed when used in conjunction with the switching circuitry and the NM gamma probe (S5).

### 3.3. Integrated Gamma-Ultrasound Probe

The gamma and US probes were combined as shown in Figure 4. The water tank was scanned with a 1-mm step size along the *x*-axis across the center line of the two targets (radiation source and glass slab) at a fixed *y*-axis position. The data were acquired using the oscilloscope for a fixed one-minute time period at each scanning step. The post-processing algorithm was applied in MATLAB to process the individual data of each module.

**Figure 6:**
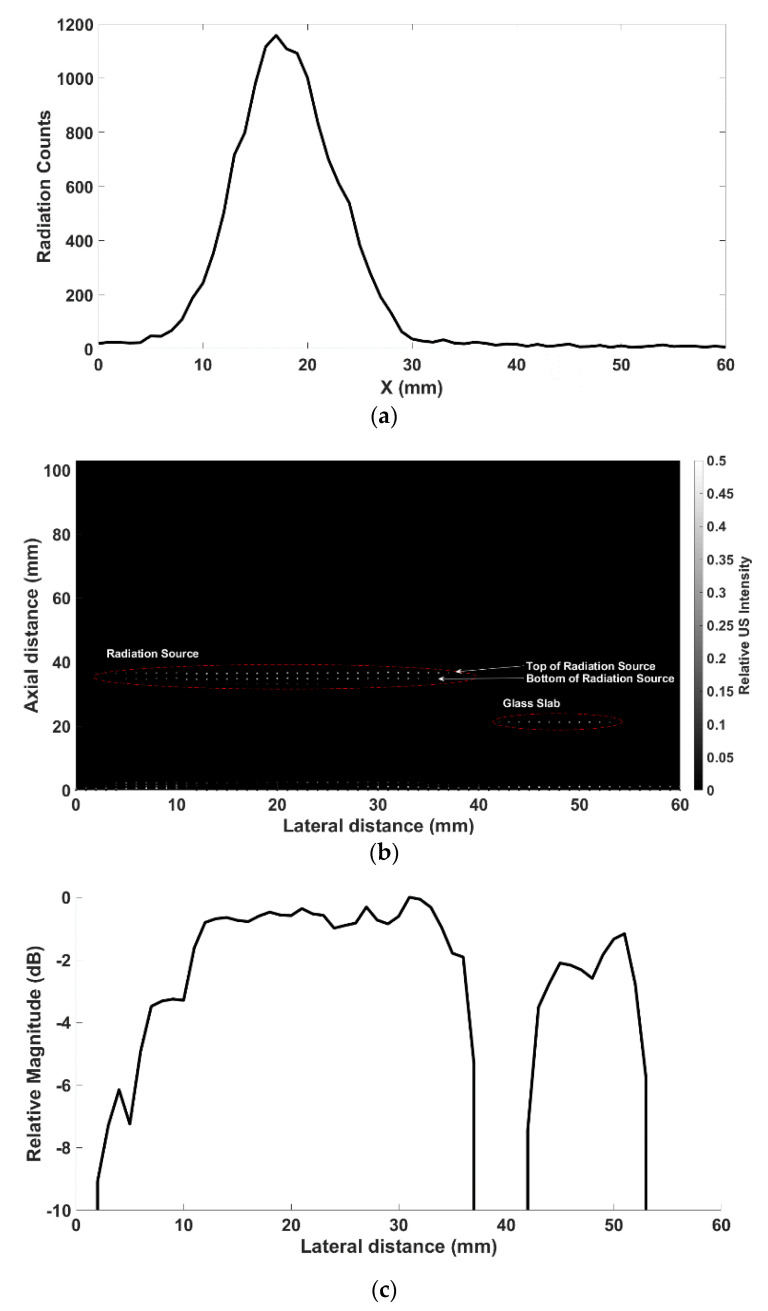

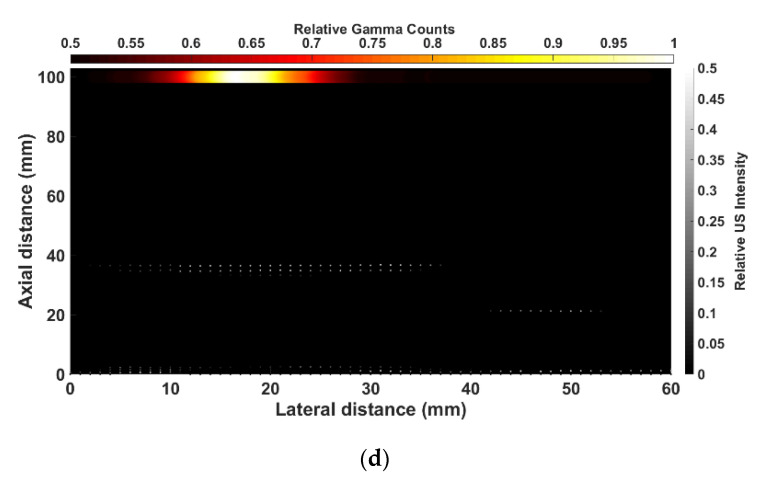
(**a**) Gamma count profile across the water tank as acquired using the standalone gamma probe. The *x*-axis location of the radiation source (i.e., molecular information in NM images) can be acquired from the profile, albeit without depth information. (**b**) 2D US image across the two targets placed in the water tank. The position (*x*-axis and depth) of the two targets are clearly visible, while the system was unable to distinguish between the radiation source and the glass slab. (**c**) Relative magnitude achieved by echo signal with the proposed system. Two targets (radiation source and glass slab) are clearly distinguished. (**d**) Union of two types of data (US-gamma) where the overlap of the images corresponds to the position of the radiation source (molecular information and structural information). The red and white bar represents the relative gamma radiation counts at each point, while the black and white bar represents the relative US echo signal intensity.

In terms of the NM gamma probe, counts that fall within the Co-57 110–132 KeV full energy peak were used to recreate the one-dimensional (1D) counts map (transmission profile). The NM gamma probe count (cps) profile for each x-position was acquired and drawn, as shown in Figure 6a. With this profile, it was possible to identify the location of the radiation source along the *x*-axis.

Similarly, the acquired echo US data at each step was used to obtain a US two-dimensional (2D) depth profile for each x-position across the water tank (Figure 6b). This US image provided the x-z position of both the radiation source and the glass slab.

After correcting for the distance between the two probes, the data profile of each module (US-NM) was plotted, as shown in Figure 6d. The one-dimensional gamma count profile in Figure 6d shows the line profile of each modality (US-NM). The combined US-gamma profile not only revealed the hotspot of the radiation counts but, also, the depth position of the radiation source, while, if required, the thickness information can also be extracted.

## 4. Discussion

In this study, we proposed an integrated front-end circuit for a hybrid US-gamma probe. The proposed circuit not only reduced the number of readout channels to compact the electronics but, also, reduced the cost of the data acquisition system. Several experiments were performed to assess the validity of the proposed method. In this proof-of-concept study, we used a single US probe and NM gamma detector (probe). The experimental results demonstrated that the use of the proposed integrated front-end circuit had little effect (except at high US PRFs) on the system’s performance.

The energy resolution and count rate are among the most common and vital parameters for assessing a gamma probe. Therefore, we compared the energy resolution of the gamma probe alone with that of the proposed switching circuit. Here, there was no degradation in terms of FWHM in both cases. Energy resolutions of 18.4% and 17.4% were achieved for the systems without and with the switching circuit (setups S1 and S2), respectively. In fact, the energy resolution was seen to improve when the switching circuit and US probe were working simultaneously, while this could be attributed to random measurement fluctuations. However, the count rate of the gamma probe was dependent on the PRF of the US system, as was expected. As the PRF of the US system was increased, the count rate of the gamma probe decreased, as Table 1 shows. At a 1-KHz PRF, which is a typical value used in medical US imaging, a slight count loss (17.9%) was observed. This count loss is essentially dependent on the US duty factor, and the count loss could be decreased by shortening the US pulse widths. In nuclear medicine, although a high count rate (detection efficiency) is desirable to reduce statistical uncertainties, nuclear medicine gamma probes often operate at low detector efficiency that varies from about 0.03~0.2% (at a 3-cm distance), depending on the manufacturer [28], implying that gamma probes are relatively robust to count rate performance. That said, this count loss can be compensated by increasing detector size or scan time.

On the US system side, our proposed readout method sufficiently improved the signal-to-noise ratio (SNR) compared with the raw US signal. The proposed circuit also provide improved peak-to-peak voltages of the echo signals in comparison with the raw US signal. With the switching circuit working in conjunction with the gamma probe, a small degradation was observed. While this is not expected to affect the overall performance, we conjecture that the switching IC’s circuitry (internal resistance and parasitic capacitance) slightly affects the signal quality.

The NM gamma probe data alone generally suffers from relatively poor spatial resolution and a lack of depth information, while a US image can provide accurate two-dimensional/three-dimensional localization of an object of interest but is unable to provide the molecular information that NM can offer. However, as demonstrated in this study, the registration of both US and NM gamma information enables an accurate position localization of the radiation source (Figure 6d). The mismatch between the actual target sizes and that in Figure 6d is due to the wide lateral ultrasound beam width and can be mitigated using a smaller transducer or focusing.

That is, we were able to identify the exact position of the object of interest (radiation source), along with the depth information, with the US providing structural information (the glass slab and the sealed radiation source in this study) while the NM provided molecular information (radiation source). These two forms of complementary information can be highly useful in various areas of medical science, such as intraoperative surgical probing to accurately identify and localize tumor tissues, while the system could also be applied in nondestructive testing (NDT), such as the measurement of concrete/pipe thicknesses [19,29].

The proposed hybrid detector and electronics can be extended to imaging systems. The number of readout channels in an imaging ultrasonic probe ranges from tens to hundreds of channels, comparable to the number of channels in a gamma camera. Research projects are underway to integrate US probe and NM detectors into a single imaging module [9], and such a system could utilize the analog front-end proposed in this paper, although additional components such as time-gain compensation would be required following the analog front-end described in this paper.

## 5. Conclusions

In this proof-of-concept study, we proposed an integrated front-end data acquisition circuit for simultaneous readouts from hybrid US-gamma probes to acquire structural and molecular information. The results indicated that the circuit could provide a compact and cost-effective solution for a hybrid US-NM hybrid probe with relatively small performance degradation. The applications of such a system include medical science (medical imaging and intraoperative probes) and NDTs. Future studies will be focused on integrating both a US transducer and a gamma detector into a single (or array) probe, along with the proposed circuit, to provide a compact and real-time solution for a highly integrated hybrid probe that can be used in various applications.

## Figures and Tables

**Table 1:** Summary of the gamma probe count rate performance at various PRFs of the US transmitter circuit.

Pulse Repetition Frequency (kHz)	Counts Per Second (cps)	Relative CPS Loss Percentage (%)
0	275.8	0.0
0.5	248.7	9.8
1	226.5	17.9
2	163.5	40.7
3	106.6	61.3
4	52.4	81.0

**Table 2:** Summarized performance parameters of the US probe via the acquisition of data with various front-end circuits. SNR: signal-to-noise ratio.

Circuit Type/Data Setup	Echo (RX) SNR (dB)	Echo (RX) Peak-to-Peak Voltage (V)	−6dB Bandwidth (%)	Center Frequency (MHz)
Raw RX signal (S3)	10.02	0.08	44.7	9.5
Custom front-end US circuit (S4)	14.89	1.11	27.8	9.0
Integrated US-gamma readout (S5)	13.12	0.97	25	9.0

## Data Availability

The data presented in this study are available on request from the corresponding author.
